# A retrospective analysis of the prevalence of hypophosphatemia and hypocalcemia after intravenous iron polymaltose in the inpatient setting

**DOI:** 10.1093/jbmrpl/ziaf103

**Published:** 2025-12-06

**Authors:** Lauren C Burrage, Stacey Llewellyn, Leanne Foyn, Carel Pretorius, Syndia Lazarus

**Affiliations:** Department of Endocrinology and Diabetes, Royal Brisbane and Women’s Hospital, Brisbane, QLD 4029, Australia; Department of Endocrinology and Diabetes, Sunshine Coast University Hospital, Birtinya, QLD 4575, Australia; Faculty of Medicine, The University of Queensland, Brisbane, QLD 4006, Australia; QIMR Berghofer, Brisbane, QLD 4006, Australia; Department of Chemical Pathology, Pathology Queensland, Brisbane, QLD 4029, Australia; Faculty of Medicine, The University of Queensland, Brisbane, QLD 4006, Australia; Department of Chemical Pathology, Pathology Queensland, Brisbane, QLD 4029, Australia; Department of Endocrinology and Diabetes, Royal Brisbane and Women’s Hospital, Brisbane, QLD 4029, Australia; Faculty of Medicine, The University of Queensland, Brisbane, QLD 4006, Australia

**Keywords:** hypocalcemia, hypophosphatemia, i.v. iron infusion, iron polymaltose, ferric carboxymaltose, FGF23

## Abstract

Hypophosphatemia occurring after ferric carboxymaltose or iron polymaltose infusion is common and has been well-characterized. Post-iron infusion hypocalcemia has been documented in case reports, but there is no data on the prevalence of hypocalcemia developing after i.v. iron infusion. In this retrospective cohort study, we sought to characterize integrated daily changes in serum phosphate and corrected calcium levels before and after iron infusion in a real-world inpatient setting. Inpatients who received i.v. iron polymaltose at the Royal Brisbane and Women’s Hospital (Queensland, Australia) between January 2020 and September 2021 were included. We extracted all results for serum phosphate and corrected calcium levels for 21 d before and after iron infusion. A total of 741 patients with 8272 blood samples were included. The serum phosphate concentration reduced by an average of 30.6% (95% CI 28.2-32.9) in the 5 d following infusion. Serum phosphate reached a nadir at day 5 before incrementing, but still remained below baseline by the end of the study period. Conversely, serum corrected calcium levels increased within 1 d of iron polymaltose infusion and then dropped over the following 5 d. There was a significant increase in the prevalence of hypophosphatemia developing after iron polymaltose infusion (34% post-infusion vs 8% pre-infusion, *p*-value < .001). Hypophosphatemia occurred most commonly within the first week after iron infusion, whereas hypocalcemia was more frequently a later occurrence. Our results have enhanced the understanding of the day-to-day biochemical changes occurring after iron infusion as well as the prevalence and timing of post-iron infusion hypophosphatemia and hypocalcemia.

## Introduction

Intravenous iron infusions are increasingly used to treat iron deficiency because of their convenience, tolerability, and efficacy compared with oral iron preparations.^1^ The i.v. formulations available in Australia include iron polymaltose (Ferrosig), ferric carboxymaltose (Ferinject), iron sucrose (Venofer), and ferric derisomaltose (Monofer).^2^^,^^3^ Iron polymaltose is the most common formulation used in Queensland hospitals,^3^ although ferric carboxymaltose is popular in the outpatient setting due to ease of administration and lower risk of hypersensitivity reactions.^1^^,^^4^ Hypersensitivity reactions and extravasation were once considered to be the primary complications of i.v. iron infusion,^1^^,^^3^ but post-iron infusion hypophosphatemia is now an increasingly recognized complication.

The first case report of hypophosphatemia developing after iron infusion was published in 1982.^5^ The same authors subsequently demonstrated in a prospective study that i.v. iron oxide caused a reduction in tubular reabsorption of phosphate, but the underlying mechanism was not understood.^6^ More than 2 decades later, Schouten et al.^7^ published the first case report of iron polymaltose-induced hypophosphatemia associated with FGF23 elevation, and prospectively demonstrated that i.v. iron polymaltose lead to a significant rise in intact FGF23 levels.^8^ FGF23 is a hormone secreted by osteocytes that controls phosphate balance by down-regulating the sodium-phosphate co-transporter 2a and 2c channels in the proximal renal tubule.^9^^,^^10^ This inhibits phosphate reabsorption and leads to phosphaturia. FGF23 also reduces 1-alpha-hydroxylase expression and thus 1,25(OH)2D3 (calcitriol) production, thereby reducing intestinal absorption of phosphate and calcium.^9^ This leads to a compensatory secondary hyperparathyroidism, which can then exacerbate phosphaturia.^10^^,^^11^

Parenteral iron formulations are composite nanoparticles, where iron oxides are complexed with carbohydrate moieties (such as polymaltose or carboxymaltose).^1^^,^^10^ However, the carbohydrate complexes within certain iron formulations have the unintended consequence of preventing degradation of FGF23.^1^^,^^9^^,^^11^ The exact mechanism of this process is not fully elucidated, but may be due to interference of the specific carbohydrate complex with post-translational modifications that would usually protect FGF23 from cleavage.^10^^,^^12^ Furthermore, it has been shown that iron deficiency itself leads to upregulation of FGF23 transcription and cleavage,^13^ although these changes occur in parallel such that the state of iron deficiency itself does not lead to hypophosphatemia.^10^ This gives credence to the “two hit” hypothesis, where the combination of increased FGF23 transcription (as a consequence of iron deficiency) in the setting of reduced FGF23 cleavage (as a consequence of the parenteral iron formulation) leads to excess levels of intact FGF23 and the cascade of biochemical abnormalities described above.^9–12^ This is now recognized as the principal mechanism that causes hypophosphatemia after iron infusion.

The prevalence of post-iron infusion hypophosphatemia can be as high as 92%,^14^^,^^15^ although most studies report a prevalence between 27%-75%.^4^^,^^13^^,^^16–21^ The wide range reflects inconsistent definitions of hypophosphatemia and heterogeneity in study design and population. The nadir in serum phosphate concentration typically occurs after 1-2 wk but the duration is less well characterized.^8^^,^^13^^,^^16^^,^^17^^,^^21^^,^^22^ Retrospective studies have suggested a median duration of 6-12 wk,^16^^,^^19^ but case studies have reported that hypophosphatemia can last for several months,^23^ or sometimes years.^24^ The strongest risk factor for developing post-infusion hypophosphatemia is the type of iron formulation that is administered; several studies have shown that hypophosphatemia is significantly more common with ferric carboxymaltose compared with ferumoxytol,^17^^,^^25^ or ferric derisomaltose.^4^^,^^16^^,^^18^^,^^20^^,^^21^ Other risk factors include preserved renal function, repeated doses of i.v. iron, baseline serum phosphate level, low body weight, and concomitant use of anti-resorptives.^10^^,^^17^^,^^19^^,^^22^ Pre-existing 25OHD deficiency and secondary hyperparathyroidism have also been proposed as risk factors,^11^^,^^26–28^ although this was not supported by a large randomized controlled trial.^17^

Hypophosphatemia that developed following iron infusion was originally considered to be an insignificant phenomenon because early studies did not document adverse clinical sequelae.^9^^,^^11^^,^^22^ However, case reports have shown that the complications of hypophosphatemia can be serious, particularly in those who receive repeated iron infusions or have other risk factors.^23^^,^^26–29^ Complications include prolonged hospital admission due to persistent requirement for i.v. phosphate,^28^^,^^30^^,^^31^ osteomalacia (characterized by bone pain and fractures),^7^^,^^23^^,^^27–29^^,^^32^ and respiratory compromise.^33^^,^^34^ Treatment is typically with phosphate replacement and commencement of calcitriol, although clinicians should be aware of the possible increased risk of nephrolithiasis with calcitriol use in this setting.^35^ If there is an ongoing iron infusion requirement, it is recommended to switch to an alternative formulation with a lower risk of hypophosphatemia.^10^^,^^11^^,^^32^ Clinicians should maintain a low threshold to check serum phosphate concentration in symptomatic individuals after iron infusion and especially in patients who receive repeated doses of i.v. iron or have other risk factors.

Although post-iron infusion hypophosphatemia has been well defined, we are not aware of any studies that have characterized the prevalence of post-iron infusion hypocalcemia. The rationale to perform this study was based on clinical experience managing 2 cases of symptomatic, severe hypocalcemia that occurred between 7 and 10 d after i.v. iron polymaltose infusion and corresponded with hypophosphatemia, reduced serum calcitriol concentration, and markedly elevated levels of FGF23 (Table 1). Both patients had additional risk factors for hypocalcemia, specifically 25OHD deficiency in the first case and prior denosumab administration in the second case. We hypothesized that hypocalcemia is an under-recognized complication of i.v. iron infusion that, similar to hypophosphatemia, develops in the setting of excess FGF23.

**Table 1:** Two cases of hypocalcaemia developing after iron polymaltose infusion.

	Case 1		Case 2		Reference range
	Baseline	3 mo	Baseline	3 mo	
**Corrected calcium**	**1.80**	2.28	**1.82**	2.38	2.10-2.60 mmol/L
**Phosphate**	**0.34**	1.04	**0.59**	1.62	0.75-1.50 mmol/L
**Creatinine**	72	68	50	67	36-73 μmol/L
**eGFR**	78	84	>90	87	>60 mL/min/1.73 m^2^
**PTH**	**35**	**11**	**47**	5.7	1-7 pmol/L
**25OHD**	**36**	62	81	**37**	50-150 nmol/L
**Calcitriol**	**36**	124	-	-	48 − 190 pmol/L
**FGF-23**	**414**	-	**591**	-	23-95 ng/L

Biochemical parameters at baseline and after 3 mo of 2 cases of hypocalcemia occurring after iron infusion. Figures highlighted in bold indicate abnormal results. Dash indicates missing data.

## Aims

The primary aim was to evaluate whether there is a significant change in serum phosphate or corrected calcium levels before and after i.v. iron polymaltose in the inpatient setting. Secondary aims were to determine whether the prevalence of hypophosphatemia (serum phosphate level < 0.75 mmol/L) or hypocalcemia (serum corrected calcium level < 2.10 mmol/L) increased after iron infusion, to elucidate the timing in which hypophosphatemia and hypocalcemia developed after iron infusion, and the impact of 25OHD deficiency on these events.

## Materials and methods

### Study design

This study was a single-center retrospective cohort study of all patients who received i.v. iron polymaltose in the inpatient setting at the Royal Brisbane and Women’s Hospital (Queensland, Australia) from January 2020 to September 2021. Ethics approval was obtained through the Royal Brisbane and Women’s Hospital (EX/2021/QRBW/80764).

### Data collection

Inpatients who received i.v. iron infusions at the Royal Brisbane and Women’s Hospital between January 2020 and September 2021 were identified via pharmacy records. Only patients who received i.v. iron polymaltose were included due to low numbers of individuals who received ferric carboxymaltose within the inpatient setting. In order to establish the timing of hypophosphatemia and hypocalcemia after iron infusion in this population, we first identified all patients who developed hypophosphatemia or hypocalcemia on Pathology Queensland blood tests within 12 mo from the date of iron infusion. The nadir serum phosphate and corrected calcium level, timing of the nadir relative to the day of infusion, and duration of episode were recorded. We established that the majority of episodes occurred within 21 d following iron infusion. We therefore extracted all available results for serum phosphate and corrected calcium levels for up to 21 d prior and 21 d after iron infusion for each patient and examined the absolute change in serum phosphate and corrected calcium levels pre- and post-iron polymaltose infusion, as well as the number of patients who developed hypophosphatemia or hypocalcemia within this time frame. If there were multiple blood tests on a single day, we only included the first sample of the day. Blood tests on the day of infusion were excluded as these could not be reliably classified as pre- or post-infusion. 25OHD levels were recorded if available within 3 mo before or 1 wk after iron infusion. Individuals with an estimated glomerular filtration rate <20 mL/min/1.73 m^2^ were excluded. Patients who received more than 1 iron infusion within the defined time frame had only the results before and after the first infusion included.

### Statistical analysis

Patient characteristics were summarized with frequencies and percentage for categorical data and median (interquartile range [IQR], range) for non-normally distributed continuous data. To assess changes over time in serum phosphate and corrected calcium levels pre-iron polymaltose infusion and post-iron polymaltose infusion within and after 5 d, lognormal mixed effects models using segmented regression approach were run,^36^^,^^37^ with the glmmTMB package.^38^ Time was considered in reference to day of iron infusion, with samples on the day of iron infusion (day 0) excluded from the analysis as they were unable to be reliably categorized as pre- or post-infusion. The model included 2 phase indicator variables: ${\mathrm{Phase}}_{\mathrm{post},i}$, given a value of 0 for pre-iron infusion samples and 1 for post-iron infusion samples; and ${\mathrm{Phase}}_{\mathrm{post}>5,i}$, given a value of 0 for pre-iron infusion samples and samples taken in the first 5 d following iron infusion, and a value of 1 for samples more than 5 d following iron-infusion. Three time variables were included in the model. A standard time variable covering the full study period from day −21 to 21 d from iron infusion (${\mathrm{Time}}_{ij}$)_,_ and 2 time-by-phase interaction terms, transformed to sequentially start at 1 within each phase as follows: ${\mathrm{Time}}_{\mathrm{Post}, ij}={\mathrm{Time}}_{ij}\times{\mathrm{Phase}}_{\mathrm{post},i}$ and ${\mathrm{Time}}_{\mathrm{Post}>5\ \mathrm{d}, ij}=\left({\mathrm{Time}}_{ij}-5\right)\times{\mathrm{Phase}}_{\mathrm{post}>5\ \mathrm{d},i}$. Time was scaled from day to week to aid in interpretation. Random intercepts overall, and for each of the phase indicator variables were included at the patient level (${u}_{ik}$). Insufficient data was available at the patient level to allow the inclusion of random slopes. The model residuals, ${\varepsilon}_{ijk}$ were assumed to be independent and identically distributed (i.d.d.) with a mean of 0 and variance of σ^2^ on the log scale. The model can be described as follows for patient $i$($i=1,\dots, N$) at time $j\ \left(j=-21,\dots, 21\ \mathrm{d}\right)$and at *k* phase $\left(k=\mathrm{pre},\kern0.5em \mathrm{post},\kern0.5em \mathrm{post}>5\ \mathrm{d}\right)$, where ${Y}_{ij}$ is the measure (phosphate/corrected calcium in mmol/L):


\begin{align*} \mathrm{Log}\left({Y}_{ij}\right)=&\,{\beta}_0+{\beta}_1{\mathrm{Phase}}_{\mathrm{post},i}+{\beta}_2{\mathrm{Phase}}_{\mathrm{post}>5,i}+{\beta}_3{\mathrm{Time}}_{ij}\\ &+{\beta}_4{\mathrm{Time}}_{\mathrm{post}, ij}+{\beta}_5{\mathrm{Time}}_{\mathrm{post}>5\ \mathrm{d}, ij}+{\varepsilon}_{ij k}+{u}_{ik}. \end{align*}

Adequacy of the model fit was evaluated using DHARMa residuals, a simulation-based residuals diagnostic approach used to indicate potential model misspecification or deviations from model assumptions.^39^ Post-hoc contrasts for comparisons of estimated marginal means at selected time-points were performed using the emmeans package, with a false discovery rate correction to account for multiple comparisons.^40^

Differences in the prevalence of hypophosphatemia and hypocalcemia before and after iron polymaltose infusion were assessed using McNemar’s test. Chi-square or Fisher’s exact test was used to evaluate the impact of vitamin D deficiency (25OHD level < 50 nmol/L) on the prevalence of hypophosphatemia and hypocalcemia. Fisher’s exact test was used if more than 20% of the expected cell counts were <5, otherwise Chi-square test was used. Statistical significance was set at a *p*-value < .05 (2-sided). R statistical package version 4.3.1 was used for all analyses.^41^

## Results

**Figure 1:**
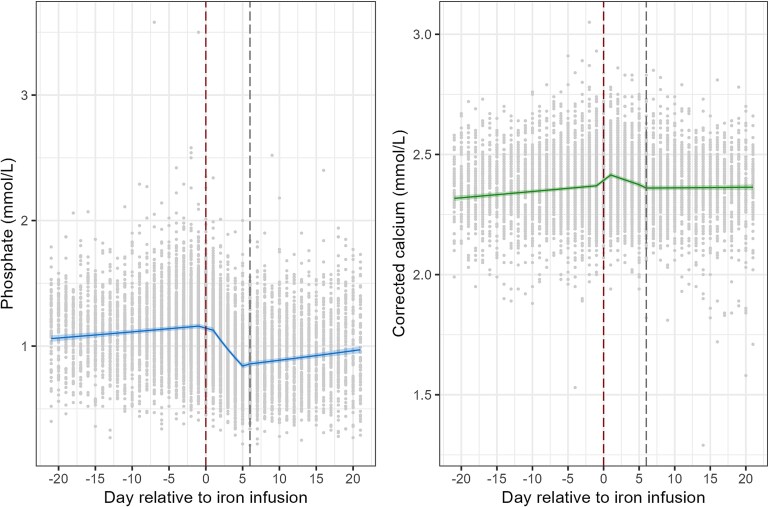
Serum phosphate and corrected calcium levels over time following iron polymaltose infusion. Predicted mean estimates (95% CI) from the lognormal mixed effects model are displayed, back transformed to the original scale. Observed patient data are shown as dots. Day of iron infusion is indicated by the vertical red (or left) dashed line, and day 6 (start of the third phase in the model) is indicated by the vertical gray (or right) dashed line.

There were 741 patients who received iron polymaltose infusion with a total of 8272 samples taken during the study period. Patient characteristics are summarized in Table 2. Changes in serum phosphate and corrected calcium levels over time are described below and depicted in Figure 1. A summary of patient measures for each study day is provided in Table 3. The full model interpretation and summaries of results for the iron polymaltose lognormal mixed effect models for phosphate and corrected calcium levels over time (Table SS1) are displayed in the Appendix.

**Table 2:** Patient characteristics summary.

**Characteristic**	**Iron polymaltose** ***N* = 741**
**Gender (%)**	
**Female**	364 (49)
**Male**	376 (51)
**Age (yr)**	67 (48, 79) [16-104]
**Patients sampled on day of iron infusion (%)**	589 (79)
**No. samples/patient:**	
**Pre-iron infusion**	4 (2, 7) [1-21]
**Post-iron infusion**	4 (2, 8) [1-21]
**Post-iron infusion ≤ 5 d**	3 (1, 4) [0-5]
**Post-iron infusion > 5 d**	1 (0, 4) [0-16]

*n* (%); median (IQR) [range].

**Table 3:** Daily measures summary for serum phosphate and corrected calcium levels for patients treated with iron polymaltose (*N* = 8272 samples, for *N* = 741 patients).

**Day relative to iron infusion**	**Phosphate**			**Corrected calcium**	
	***N***	**Mean (SD)**		***N***	**Mean (SD)**
**−21**	74	1.15 (0.27)		74	2.36 (0.12)
**−20**	75	1.15 (0.26)		74	2.38 (0.11)
**−19**	83	1.16 (0.27)		83	2.37 (0.13)
**−18**	83	1.10 (0.24)		84	2.35 (0.13)
**−17**	94	1.14 (0.27)		93	2.36 (0.13)
**−16**	86	1.16 (0.28)		86	2.37 (0.13)
**−15**	101	1.12 (0.27)		101	2.36 (0.13)
**−14**	104	1.12 (0.26)		104	2.36 (0.14)
**−13**	112	1.11 (0.26)		110	2.36 (0.13)
**−12**	110	1.10 (0.26)		109	2.35 (0.13)
**−11**	125	1.10 (0.28)		124	2.36 (0.13)
**−10**	132	1.09 (0.29)		130	2.36 (0.15)
**−9**	157	1.12 (0.29)		157	2.37 (0.13)
**−8**	167	1.15 (0.26)		167	2.38 (0.12)
**−7**	205	1.18 (0.32)		205	2.38 (0.12)
**−6**	229	1.16 (0.27)		229	2.37 (0.13)
**−5**	259	1.16 (0.27)		259	2.37 (0.12)
**−4**	323	1.18 (0.29)		323	2.37 (0.13)
**−3**	397	1.18 (0.28)		397	2.37 (0.12)
**−2**	521	1.17 (0.28)		521	2.37 (0.12)
**−1**	612	1.18 (0.28)		612	2.37 (0.12)
**0** ^**a**^	589	1.17 (0.27)		589	2.38 (0.11)
**1**	543	1.16 (0.26)		543	2.41 (0.12)
**2**	450	1.05 (0.25)		449	2.42 (0.11)
**3**	373	0.94 (0.26)		373	2.40 (0.11)
**4**	330	0.91 (0.27)		331	2.39 (0.11)
**5**	295	0.87 (0.29)		295	2.38 (0.13)
**6**	249	0.87 (0.28)		249	2.37 (0.12)
**7**	236	0.88 (0.27)		236	2.37 (0.12)
**8**	192	0.91 (0.29)		192	2.37 (0.13)
**9**	169	0.89 (0.28)		169	2.37 (0.13)
**10**	155	0.90 (0.30)		155	2.39 (0.12)
**11**	155	0.91 (0.28)		154	2.38 (0.13)
**12**	151	0.91 (0.31)		149	2.37 (0.13)
**13**	141	0.93 (0.30)		140	2.36 (0.13)
**14**	150	0.91 (0.29)		140	2.36 (0.16)
**15**	101	0.91 (0.26)		101	2.35 (0.14)
**16**	108	0.99 (0.32)		108	2.37 (0.15)
**17**	96	0.94 (0.28)		96	2.36 (0.14)
**18**	94	1.00 (0.35)		94	2.36 (0.14)
**19**	92	0.99 (0.35)		92	2.37 (0.15)
**20**	73	0.99 (0.36)		73	2.34 (0.20)
**21**	80	0.99 (0.36)		80	2.35 (0.14)

^a^Summary of patient measures presented at day 0 (day of iron infusion) not included in modeling.

### Change in serum phosphate levels pre- and post-iron polymaltose infusion

Prior to iron infusion, phosphate levels increased by an average of 3.2% (2.4%-4.0%)/wk, exp(beta) = 1.032, 95% CI 1.024-1.040, *p*-value < .001, with predicted mean level 21 d prior to iron infusion of 1.06 mmol/L (95% CI 1.03-1.09 mmol/L) and 1.16 mmol/L (95% CI 1.14-1.18 mmol/L) 1 d prior. One day after iron polymaltose infusion, there was a 2.8% reduction (95% CI 0.9%-4.6%) in phosphate levels compared to 1 d prior to infusion (ratio 0.972 [95% CI 0.954-0.991], *Z*-ratio = −2.91, *p*-value = .004). In the 5 d immediately following iron polymaltose infusion, phosphate levels decreased on average by 30.6% (95% CI 28.2%-32.9%)/5 d, with the average predicted phosphate levels decreasing from 1.13 mmol/L (95% CI 1.11-1.15 mmol/L) 1 d post-iron infusion to 0.84 mmol/L (95% CI 0.82-0.86 mmol/L) 5 d post-iron infusion, ratio 0.75 (95% CI 0.73-0.72), *Z*-ratio = −21.65, *p*-value < .001. In the period more than 5 d post-iron polymaltose infusion, there was no evidence of a consistent linear change in phosphate levels with an average increase of 5.9% (95% CI −1.1% to 13.4%)/wk. However, direct comparisons of the average predicted phosphate levels 6 d post-iron infusion and 21 d post-iron infusion showed a 13% (95% CI 10%-17%) increase from 0.86 mmol/L (95% CI 0.84-0.88 mmol/L) to 0.97 mmol/L (95% CI 0.94-1.00 mmol/L) respectively, ratio 1.13 (95% CI 1.10-1.17), *Z*-ratio = 7.45, *p*-value < .001. At the end of the study period, average predicted phosphate levels were still 16.2% (95% CI 13.6%-18.8%) lower compared with day 1 prior to iron polymaltose infusion, ratio 0.838 (95% CI 0.812-0.864), *Z*-ratio = −11.21, *p*-value < .001.

### Change in serum corrected calcium levels pre- and post-iron polymaltose infusion

Prior to iron infusion, corrected calcium levels increased by an average of 0.8% (0.6%-0.9%)/wk, exp(beta) = 1.008, 95% CI 1.006-1.009, *p*-value < .001, with predicted mean levels 21 d prior to iron infusion of 2.32 mmol/L (95% CI 2.31-2.33 mmol/L) and 2.37 mmol/L (95% CI 2.36-2.38 mmol/L) 1 d prior. After iron polymaltose infusion, there was an increase in corrected calcium levels from 1 d prior to 1 d post-iron infusion of 1.9% (95% CI 1.6%-2.2%; ratio 1.019 (95% CI 1.016-1.022), *Z*-ratio = 11.13, *p*-value < .001). In the 5 d immediately following iron polymaltose infusion, corrected calcium levels decreased on average by 2.1% (95% CI 1.6%-2.6%)/5 d. The average predicted corrected calcium levels decreased from 2.42 mmol/L (95% CI 2.41-2.42 mmol/L) 1 d post-iron infusion to 2.37 mmol/L (95% CI 2.36-2.38 mmol/L) 5 d post-iron infusion, ratio 0.983 (95% CI 0.979-0.987), *Z*-ratio = −8.20, *p*-value < .001. In the period more than 5 d post-iron polymaltose infusion, there was no evidence of a change in corrected calcium levels over time with an average increase of 0.1% (95% CI −1.0% to 1.1%)/wk. There was also no significant difference in the average predicted corrected calcium levels at 1 d prior (2.37 mmol/L, 95% CI 2.31-2.33 mmol/L) compared with 21 d post-iron polymaltose infusion (2.36 mmol/L, 95% CI 2.35-2.38 mmol/L), ratio 1.00 (95% CI 0.99-1.00), *Z*-ratio = −0.95, *p*-value = .43.

### Prevalence of hypophosphatemia and hypocalcemia

The number of patients who developed hypophosphatemia and hypocalcemia before and after iron polymaltose infusion is summarized in Table 4. There was a significant increase in the prevalence of post-iron infusion hypophosphatemia: 8% (*n* = 59) of patients had hypophosphatemia that only occurred prior to iron infusion, 34% (*n* = 251) of patients had hypophosphatemia that only occurred after infusion and 11% (*n* = 84) of patients had hypophosphatemia that occurred both before and after infusion (*p*-value < .001). There was no significant difference in the prevalence of hypocalcemia: 5% (*n* = 36) of patients had hypocalcemia prior to iron infusion, 3% (*n* = 26) of patients developed hypocalcemia after iron polymaltose infusion and 1% (*n* = 6) had hypocalcemia both before and after iron infusion (*p*-value = .253).

**Table 4:** The number of patients who developed hypophosphatemia or hypocalcemia relative to administration of i.v. iron polymaltose infusion.

**Iron Polymaltose and Hypophosphatemia**				
	Patients without hypophosphatemia	Patients who only had pre-infusion hypophosphatemia	Patients who only had post-infusion hypophosphatemia	Patients with both pre- and post-infusion hypophosphatemia
**Number (%)**	347/741 (47)	59/741 (8)	251/741 (34)	84/741 (11)
				(χ²(1) = 117.7) *p*-value < .001
**Iron Polymaltose and Hypocalcemia**				
	Patients without hypocalcemia	Patients who only had pre-infusion hypocalcemia	Patients who only had post-infusion hypocalcemia	Patients with both pre- and post-infusion hypocalcemia
**Number (%)**	673/741 (91)	36/741 (5)	26/741 (3)	6/741 (1)
				(χ²(1) = 1.31) *p*-value = .253

Absolute numbers of patients (percentage). Patients without hypophosphatemia or hypocalcemia were reported in the first column. Pre-infusion hypophosphatemia and hypocalcemia refers to the number of patients who only had hypophosphatemia or hypocalcemia prior to iron infusion. Post-infusion hypophosphatemia and hypocalcemia refers to the number of patients who only developed hypophosphatemia or hypocalcemia after iron infusion. Both pre- and post-infusion hypophosphatemia refers to the number of patients who had hypophosphatemia or hypocalcemia both before and after iron infusion. *p*-values were derived by McNemar test.

**Figure 2:**
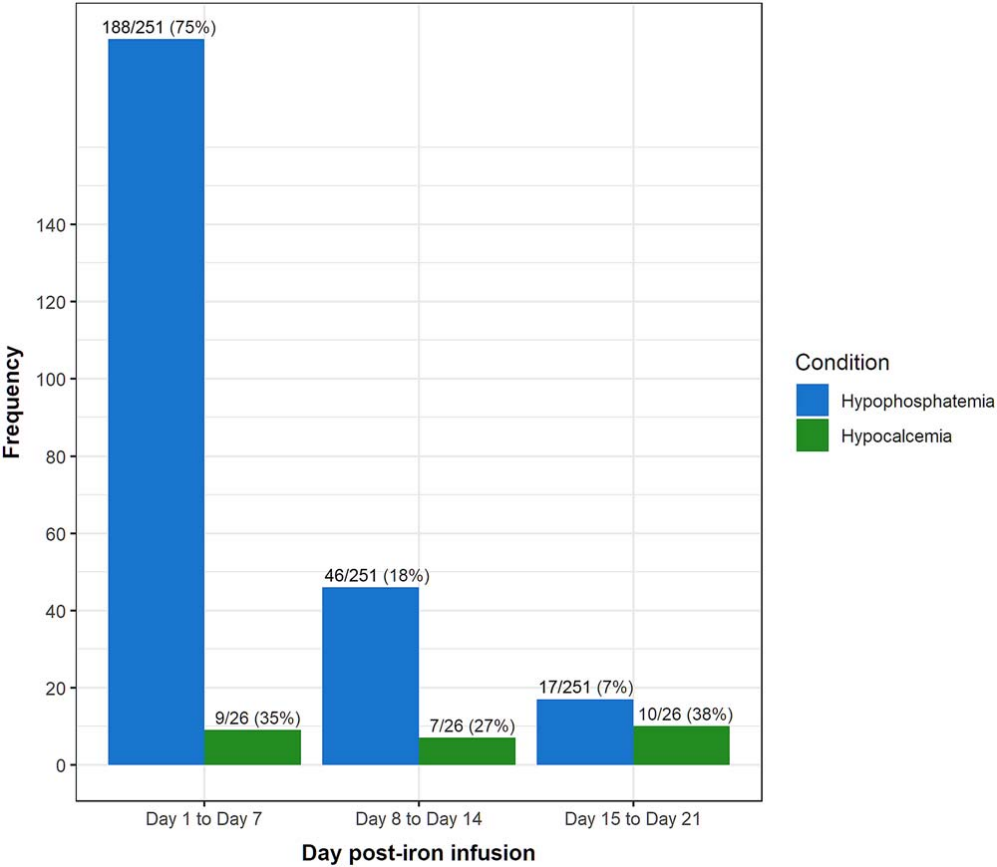
The number of patients who had the first instance of hypophosphatemia (blue, or left bars) or hypocalcemia (green, or right bars) within week 1 (days 1-7), week 2 (days 8-14) and week 3 (days 15-21) following iron polymaltose infusion.

Hypophosphatemia occurred most frequently within 7 d of iron polymaltose infusion and became less common over time, although new onset hypophosphatemia continued to develop even in the third week post-infusion (Figure 2). In contrast, the timing of hypocalcemia was similar across the 3 wk but occurred most frequently in the third week after iron infusion (Figure 2). Thirty-eight percentage of new onset hypocalcemia cases occurred within the third week post-iron infusion compared with 7% of hypophosphatemia.

### Impact of vitamin D deficiency on prevalence of hypophosphatemia and hypocalcemia

25OHD levels were available in 295 patients (39%). Seventy-eight patients were vitamin D deficient (25OHD level < 50 nmol/L) and 217 patients were vitamin D replete (25OHD level > 50 nmol/L). Vitamin D deficiency was not associated with the occurrence of hypophosphatemia (*p*-value = .092) or hypocalcemia (*p*-value = .243) before or after i.v. iron polymaltose infusion (Table 5).

**Table 5:** Impact of vitamin D deficiency on the prevalence of hypophosphatemia and hypocalcemia after iron polymaltose infusion.

**Hypophosphatemia**				
	Patients without hypophosphatemia	Patients who only had pre-infusion hypophosphatemia	Patients who only had post-infusion hypophosphatemia	Patients with both pre- and post-infusion hypophosphatemia
**Vitamin D replete (%)**	111/217 (51)	10/217 (5)	71/217 (33)	25/217 (11)
**Vitamin D deficient (%)**	30/78 (38)	3/78 (4)	28/78 (36)	17/78 (22)
				χ^2^(1) = 6.44, *p*-value = .092
**Hypocalcemia**				
	Patients without hypocalcemia	Patients who only had pre-infusion hypocalcemia	Patients who only had post-infusion hypocalcemia	Patients with both pre- and post-infusion hypocalcemia
**Vitamin D replete (%)**	202/217 (93)	6/217 (3)	6/217 (3)	3/217 (1)
**Vitamin D deficient (%)**	70/78 (90)	6/78 (8)	1/78 (1)	1/78 (1)
				*p*-value = .243

Absolute numbers of patients (percentage), per vitamin D status. As above, patients without hypophosphatemia or hypocalcemia were reported in the first column. Pre-infusion hypophosphatemia and hypocalcemia refers to the number of patients who only had hypophosphatemia or hypocalcemia prior to iron infusion. Post-infusion hypophosphatemia and hypocalcemia refers to the number of patients who only developed hypophosphatemia or hypocalcemia after iron infusion. Both pre- and post-infusion hypophosphatemia refers to the number of patients who had hypophosphatemia or hypocalcemia both before and after iron infusion. *p*-values derived by Chi square test for hypophosphatemia and Fisher’s exact test for hypocalcemia, based on expected cell counts.

## Discussion

To the best of our knowledge, this is the first study to examine integrated daily changes in serum phosphate and corrected calcium levels for 3 wk before and after i.v. iron infusion in a real-world inpatient setting. The temporal changes in serum phosphate concentration that we observed are similar to other large studies,^13^^,^^17^^,^^18^^,^^21^ and our analysis successfully demonstrated significant post-iron polymaltose infusion hypophosphatemia. However, the mean serum calcium concentration unexpectedly rose within 1 d post iron polymaltose infusion, and there was no significant increase in the prevalence of hypocalcemia after iron infusion.

Although changes in serum phosphate concentration post-iron infusion are well characterized, there is a less complete understanding within existing literature regarding changes in serum calcium concentration after iron infusion. Some large randomized controlled trials have documented a significant reduction in serum calcium levels by week 2 post-iron infusion, but these studies have not quantified the proportion of patients who developed hypocalcemia.^13^^,^^17^^,^^18^^,^^21^ Other, smaller, prospective trials have not demonstrated any significant change in serum calcium concentration after iron infusion.^6^^,^^8^^,^^20^^,^^42^ However, most of these studies have measured serum calcium concentrations at infrequent intervals, which might underestimate the absolute change in serum calcium levels as well as the burden of hypocalcemia. In contrast, our study captured all available samples across every day of the week for 3 wk before and after iron infusion and also quantified the prevalence of hypocalcemia.

Our study showed an increase in mean serum corrected calcium concentration within 1 d following iron polymaltose infusion. This pattern was also seen in a trial of 25 iron deficient women who received i.v. ferric carboxymaltose, whereby serum calcium increased within 24 h, decreased to a nadir at day 7 and returned to normal by day 35,^13^ and was also observed in a larger randomized control trial comparing ferric carboxymaltose and ferric derisomaltose in a population of individuals with inflammatory bowel disease.^21^ It is possible that this initial increase in calcium concentration is due to FGF23-mediated stimulation of calcium transporters in the distal renal tubule, thus facilitating renal calcium conservation.^9^^,^^43^^,^^44^ In our study, the mean predicted peak in serum calcium concentration following iron polymaltose infusion (at day 1) occurred prior to the mean predicted nadir in serum phosphate concentration (at day 5) and so we speculate that up-regulation of calcium transporters in the distal renal tubule occurs before down-regulation of the phosphate transporters takes effect in the proximal renal tubule. Similar to other studies,^13^^,^^17^^,^^18^ we observed a reduction in serum corrected calcium concentration toward the end of the study period, with serum corrected calcium concentration reaching a nadir at day 20, although only *N* = 73 patients had a sample taken on this day. The term “6H syndrome” has been coined to explain the constellation of biochemical changes seen after i.v. iron infusion: high FGF23, hyperphosphaturia, hypophosphatemia, hypovitaminosis D, hypocalcemia, and secondary hyperparathyroidism.^1^^,^^11^ This could be amended to the “7H syndrome” to include the initial increase in serum calcium concentration that we, and others,^13^^,^^21^ have observed and thus more accurately describe the cascade of biochemical changes that are seen after iron infusion. “7H syndrome” would describe the syndrome of high FGF23, hypercalcemia, hyperphosphaturia, hypophosphatemia, hypovitaminosis D, hypocalcemia, and secondary hyperparathyroidism.

Although we did not identify a significant increase in the prevalence of hypocalcemia after iron infusion in this general inpatient population, there are several published case reports that have documented the occurrence of post-iron infusion hypocalcemia, particularly in patients with underlying risk factors.^22^^,^^26^^,^^28^^,^^29^^,^^31^^,^^45–50^ We found that pre-existing 25OHD deficiency was not a risk factor for post-iron infusion hypocalcemia. However, it is likely that other risk factors are more relevant. Co-administration of anti-resorptives with i.v. iron infusion is one such risk factor—the inhibition of bone resorption and reduced calcium efflux from bone compounds the iron-infused mediated increase in FGF23 and subsequent blunted parathyroid hormone response, thus resulting in hypocalcemia.^45^^,^^47^ Recent administration of denosumab has been documented in several case reports of post-iron infusion hypocalcemia.^28^^,^^34^^,^^45–48^ The earliest cases had reported this association predominantly in patients with chronic kidney disease,^45^^,^^47^ but recent reports have also documented it in individuals without pre-existing renal impairment.^34^^,^^46^^,^^48^^,^^50^ There are also several cases of post-iron infusion hypocalcemia occurring in those who receive multiple iron infusions.^22^^,^^26^^,^^28^^,^^29^^,^^31^^,^^49^ This is likely due to a stacking effect of FGF23 and is a well-documented risk factor for post-iron infusion hypophosphatemia.^14^^,^^22^^,^^26^^,^^28^^,^^31^ Future studies should focus on evaluating rates of post-iron infusion hypocalcemia in populations who have repeated iron infusions or have had recent anti-resorptive administration. It would be particularly interesting to define a time interval during which co-administration of denosumab and iron infusion significantly increases the risk of hypocalcemia. Some authors have proposed ensuring a 1-3 mo time interval between iron infusion and denosumab administration,^34^^,^^45^^,^^46^ or empirically administering calcium supplementation for 1-2 wk after co-administration of these medications,^48^ but these recommendations are anecdotal and higher quality evidence is required.

The strengths of our study include the large number of patients, processing of blood collections at a single laboratory, and the blood results covering all available samples for all days across 3 wk before and after iron infusion.

By confirming the expected changes in serum phosphate concentration and rates of hypophosphatemia after iron infusion, we were confident in the validity of our methodology. However, the retrospective design has many inherent limitations. First, there was no standardized approach to the timing of blood sample collection in this inpatient setting. Second, we did not have access to biochemical parameters such as FGF23, calcitriol, parathyroid hormone or urinary phosphate levels, which would be useful to confirm the mechanisms underpinning the fluxes in serum phosphate and calcium levels. Third, repeated iron infusions are a common occurrence in patients with iron deficiency anemia, especially those with chronic blood loss, and we could not account for iron infusions given at other sites prior to inclusion in our study. This may have over-estimated the prevalence of pre-infusion hypophosphatemia and hypocalcemia and thus underestimated the true prevalence of post-infusion hypophosphatemia and hypocalcemia. Furthermore, patients who received multiple iron infusions at the Royal Brisbane and Women’s Hospital during the period of interest within our study only had the first instance included, but multiple infusions are expected to engender a higher risk of both hypophosphatemia and hypocalcemia. Fourth, our retrospective study could not account for all factors that might affect calcium and phosphate homeostasis in a hospitalized inpatient setting including other medical comorbidities, concurrent medication administration, and dietary intake and nutritional status. Finally, by truncating the study period at 3 wk, we may have missed further reduction in serum calcium concentration beyond this point, given that the nadir serum calcium concentration in our study occurred at day 20. However, the number of blood tests taken through the hospital pathology service more than 3 wk after iron infusion would inevitably be low and a retrospective analysis would be unlikely to capture significant results. It is also likely that the duration of altered FGF23 metabolism due to iron infusion will vary between individuals and any offset effect on serum corrected calcium concentration will be less evident once data from many individuals are pooled.

Other limitations include exclusion of ferric carboxymaltose infusions due to minimal patient numbers. This reflects prescribing practices in Queensland hospitals as ferric carboxymaltose is predominantly used in an outpatient setting. However, a large prospective trial comparing the prevalence of hypophosphatemia and hypocalcemia, specifically between iron polymaltose and ferric carboxymaltose, would be particularly informative in Australia where these are the most commonly used iron preparations. Finally, only 39% of the study population had available 25OHD concentrations and only 26% were vitamin D deficient. These low numbers may have limited conclusions regarding the impact of vitamin D deficiency on the prevalence of hypocalcemia and hypophosphatemia after iron infusion. Nevertheless, our results confirmed those of Wolf et al.^17^ who also did not find a relationship between vitamin D deficiency and rates of hypophosphatemia after iron infusion.

In conclusion, our findings have provided important characterization of the day-to-day pattern of change in both serum phosphate and corrected calcium levels post-iron infusion and have confirmed that hypophosphatemia occurs frequently after i.v. iron administration. Although there was no significant change in the prevalence of post-iron infusion hypocalcemia in this general inpatient population, our data suggests that hypocalcemia would be a later and rarer event compared to hypophosphatemia. This provides reassurance that i.v. iron infusions can be safely administered to the general inpatient population without significant risk of hypocalcemia. However, future studies should evaluate the prevalence of hypocalcemia occurring after iron infusion in a population with underlying risk factors including repeated iron infusions or prior anti-resorptive administration.

## Supplementary Material

Appendix_ziaf103

## Data Availability

The data underlying this article will be shared on reasonable request to the corresponding author.
