# Supplementary material for: A retrospective analysis of the prevalence of hypophosphatemia and hypocalcemia after intravenous iron polymaltose in the inpatient setting
Source: JBMR Plus. 2025 Dec 6;9(Suppl 5):v79–87. doi: 10.1093/jbmrpl/ziaf103 (PMC12723662; doi:10.1093/jbmrpl/ziaf103)
Supplement: Appendix_ziaf103 [file appendix_ziaf103.docx]

**Appendix**

| **Characteristic** | **Phosphate** | | |  | **Corrected Calcium** | | |
| --- | --- | --- | --- | --- | --- | --- | --- |
|  | **exp(Beta)** | **95% CI***^1^* | **p-value** |  | **exp(Beta)** | **95% CI***^1^* | **p-value** |
| Intercept,$\beta_{0}$ | 1.164 | 1.145, 1.184 | <0.001 |  | 2.372 | 2.364, 2.380 | <0.001 |
| Phase: Post, $\beta_{1}$ |  |  |  |  |  |  |  |
| No | — | — |  |  | — | — |  |
| Yes | 1.041 | 1.017, 1.066 | 0.001 |  | 1.022 | 1.018, 1.026 | <0.001 |
| Phase: Post >5d,$\beta_{2}$ |  |  |  |  |  |  |  |
| No | — | — |  |  | — | — |  |
| Yes | 1.012 | 0.982, 1.042 | 0.43 |  | 0.994 | 0.989, 0.999 | 0.010 |
| Time_,_ $\beta_{3}$ | 1.032 | 1.024, 1.040 | <0.001 |  | 1.008 | 1.006, 1.009 | <0.001 |
| Time _post,_$\beta_{4}$^ | 0.581 | 0.554, 0.609 | <0.001 |  | 0.963 | 0.956, 0.970 | <0.001 |
| Time _post >5d_ $\beta_{5}$^ | 1.767 | 1.683, 1.856 | <0.001 |  | 1.031 | 1.023, 1.038 | <0.001 |
| Random effect, $u_{\mathrm{pre}}$ (σ) | 0.111 | 0.096,0.129 |  |  | 0.033 | 0.030, 0.036 |  |
| Random effect, $u_{\mathrm{post}}$ (σ) | 0.105 | 0.095, 0.116 |  |  | 0.019 | 0.018, 0.021 |  |
| Random effect, $u_{post >5d}$ (σ) | 0.119 | 0.107, 0.132 |  |  | 0.020 | 0.018, 0.022 |  |
| *^1^* CI = Confidence Interval;  ^Estimates for Time _post ≤5d_ and Time _post >5d_ represent differences in the slope (change in over time) compared to the pre-infusion and post≤5d periods, respectively.  ***Phosphate*** - Actual slope for Time _post:_ exp(beta)=0.600, 95% CI: 0.571 – 0.629; Actual slope for Time _post >5d:_ exp(beta)=1.059, 95% CI: 0.989– 1.134; Dispersion parameter 0.200 (95% CI 0.196 – 0.204); AIC -928.566  ***Corrected calcium*** - Actual slope for Time _post:_ exp(beta)= 0.971, 95% CI: 0.964 – 0.978; Actual slope for Time _post >5d:_ exp(beta)= 1.000, 95% CI: 0.990 – 1.012; Dispersion parameter 0.073 (95% CI 0.071 – 0.074); AIC -16740.67 | | | | | | | |

Supplementary Table 1. Segmented regression mixed models for serum phosphate and corrected calcium levels before and after iron polymaltose infusion (N=8272 samples, for N=741 patients).

*Full Model.*

The segmented regression model can be described as follows for patient $i$($i=1, \ldots, N)$ at time $j (j=-21,\ldots,21 days)$and at *k* phase $(k=pre, post, post>5d)$:

$$Y_{ij} = \beta_{0} {+ \beta}_{1}{Phase}_{post,i}{+ \beta}_{2}{Phase}_{post>5, i}+\beta_{3}{Time}_{ij}+\beta_{4}{Time}_{post, ij}+\beta_{5}{Time}_{post>5d, ij}+\varepsilon_{ijk}+u_{ik}$$

Where:

$Y_{ij}=\mathrm{measure}\left( phosphate or corrected calcium \right) (mmol/L)$

${Phase}_{post,i}=\text{Post-iron infusion phase}= \left\{ \begin{matrix} 0 \\ 1 \end{matrix} \right. \begin{matrix} \text{if} Time<0 \\ \text{if} Time \geq0 \end{matrix}$

${Phase}_{post>5d,i}=\text{Phase more than 5 days post-iron infusion }= \left\{ \begin{matrix} 0 \\ 1 \end{matrix} \right. \begin{matrix} \text{if} Time\leq5 \\ \text{if} Time>6 \end{matrix}$

$${Time}_{ij}=\text{Time in days from iron infusion }(integar:-21\leq{Time}_{, ij}\leq21)$$

$${Time}_{post, ij}=\text{Time in days post-iron infusion} (integar:0\leq{Time}_{post, ij}\leq21)$$

$${Time}_{post>5d, ij}=\text{Time in days from 5 days post-iron infusion} (integar:1\leq{Time}_{post>5d, ij}\leq16)$$

$u_{ik}=$random effects, which are i.d.d. for each subject for each phase with $\alpha_{i}\sim N(0, \sigma_{\alpha}^{2})$

$\varepsilon_{ijk}$= the error term, assumed to be i.d.d. with $\varepsilon_{ijk}\sim N\left( 0, \sigma^{2} \right),$independently of the random effects

*Phosphate – full model interpretation.*

Phase 1 (pre-iron infusion)

- Average phosphate levels 21 days prior to iron infusion: 1.06mmol/L (95% CI: 1.03-1.09mmol/L).
- Average predicted phosphate levels at 1 day prior to iron infusion: 1.16mmol/L (95% CI: 1.14-1.18mmol/L).
- Phosphate levels increased by an average of 3.2% (2.4 – 4.0%) per week prior to iron infusion (exp(beta)=1.032, 95% CI 1.024-1.040, p<0.001).

Phase 2 (post-iron infusion ≤5days)

- Average predicted phosphate levels 1 day post iron infusion: 1.13mmol/L (95% CI: 1.11-1.15mmol/L).
- Average predicted phosphate levels at 5 days post iron infusions: 0.84 mmol/L (95% CI: 0.82-0.86mmol/L).
- There was evidence of a change in phosphate levels by a factor of 0.581 per week prior in the period of 5 days immediately following iron infusion, compared to the pre-iron infusion period (exp(beta)=0.581, 95% CI 0.554-0.609, p<0.001).
  - This is equivalent to a decrease on average of 30.6% (95% CI 28.2 – 32.9%) per 5 days in the period of 5 days immediately following the iron infusion (slope for phase 1 + slope for phase 2: exp(beta)=0.600, 95% CI: 0.571 – 0.629).

Phase 3 (post-iron infusion >5days)

- Average predicted phosphate levels at 6 days post iron infusions: 0.86 mmol/L (95% CI: 0.84-0.88mmol/L).
- Average predicted phosphate levels at 21 days post iron infusions: 0.97 mmol/L (95% CI: 0.94-1.00mmol/L).
- There was evidence of a difference in the change in phosphate levels by a factor of 1.767 from the period 6 days following iron infusion compared to the period immediately following iron infusion (exp(beta)=1.767, 95% CI 1.683-1.856, p<0.001).
  - This is equivalent to an increase on average of 5.9% (95% CI -1.1 – 13.4%) per week in the period from six days following iron infusion (slope for phase 1 ($\beta_{3})$+ slope for phase 2 ($\beta_{4})$ + slope for phase 3 ($\beta_{5})$: exp(beta)=1.06, 95% CI: 0.99– 1.13)

Contrasts (with FDR corrections)

- There was evidence of a 2.8% reduction (95% CI 0.9-4.6%) in phosphate levels from 1 day prior to 1 day post-iron infusion (Ratio 0.972 (95% CI 0.954-0.991), Z-ratio=-2.91, p-value=0.004).
- There was evidence of a reduction in phosphate levels from 1 day prior to 5 days post-iron infusion of 27.4% (95% CI: 25.7-29.1%) (Ratio 0.726 (95% CI 0.709-0.743), Z-ratio=-26.53, p-value<0.001).
- There was evidence of a reduction in phosphate levels from 1 day post to 5 days post-iron infusion of 25.4% (95% CI: 23.4-27.3%) (Ratio 0.746 (95% CI 0.727-0.766), Z-ratio=-21.65, p-value<0.001).
- There was evidence of a reduction in phosphate levels from 1 day prior to 21 days post-iron infusion of 16.2% (95% CI 13.6-18.8%) (Ratio 0.838 (951% CI 0.812-0.864), Z-ratio=-11.21, p-value<0.001).
- There was evidence of an increase in phosphate levels from 6 days post to 21 days post-iron infusion of 13% (95% CI 10-17%) (Ratio 1.13 (95% CI 1.10-1.17), Z-ratio=7.45, p-value<0.001).

*Corrected Calcium – full model interpretation*

Phase 1 (pre-iron infusion)

- Average corrected calcium levels 21 days prior to iron infusion: 2.32mmol/L (95% CI: 2.31-2.33mmol/L)
- Average predicted corrected calcium levels for 1 day prior to iron infusions: 2.37mmol/L (95% CI: 2.36-2.38mmol/L).
- Corrected calcium levels increased by an average of 0.8% (0.6 – 0.9%) per week prior to iron infusion (exp(beta)=1.008, 95% CI 1.006-1.009, p<0.001).

Phase 2 (post-iron infusion ≤5days)

- Average predicted corrected calcium levels 1 day post iron infusion: 2.42mmol/L (95% CI: 2.41-2.42mmol/L).
- Average predicted corrected calcium levels at 5 days post iron infusions were: 2.37 mmol/L (95% CI: 2.36-2.38mmol/L).
- There was evidence of a difference in the change over time in corrected calcium levels by a factor of 0.963 per week prior in the period of 5 days immediately following iron infusion, compared to the pre-iron infusion period (exp(beta)=0.963, 95% CI 0.956-0.970, p<0.001).
  - This is equivalent to a decrease on average of 2.09% (95% CI 1.57–2.60%) per 5 days in the period of 5 days immediately following the iron infusion (slope for phase 1${(\beta}_{3})$ + slope for phase 2($\beta_{4}$): exp(beta)=0.971, 95% CI: 0.964–0.978).

Phase 3 (post-iron infusion >5days)

- Average predicted corrected calcium levels for 6 days post iron infusions were: 2.36 mmol/L (95% CI: 2.35-2.37mmol/L).
- Average predicted corrected calcium levels for 21 days post iron infusions were: 2.36 mmol/L (95% CI: 2.35-2.38mmol/L).
- There was evidence of a difference in the change in corrected calcium levels by a factor of 1.031 (95% CI 1.023 – 1.038, p<0.001) from the period 6 days following iron infusion compared to the period immediately following iron infusion.
  - This is equivalent to an increase on average of 0.07% (95% CI -1.0–1.12%) per week in the period from six days following iron infusion (slope for phase 1 ($\beta_{3})$ + slope for phase 2 ($\beta_{4})$ + slope for phase 3 ($\beta_{5})$: exp(beta)=1.00, 95% CI: 0.99– 1.01).

Contrasts (with FDR corrections)

- There was evidence of an increase in corrected calcium levels from 1 day prior to 1 day post-iron infusion of 1.9% (95% CI 1.6-2.2%) (Ratio 1.019 (95% CI 1.016-1.022), Z-ratio=11.13, p-value<0.001).
- There was no evidence of a change in corrected calcium levels from 1 day prior to 5 days post-iron infusion (Ratio 1.00 (95% CI 0.99-1.00), Z-ratio=-1.03, p-value=0.43).
- There was evidence of decrease in corrected calcium levels from 1 day post to 5 days post-iron infusion of 1.7% (95% CI 1.3–2.1%) (Ratio 0.983 (95% CI 0.979-0.987), Z-ratio=-8.20, p-value<0.001).
- There was no evidence of a change in corrected calcium levels from 1 day prior to 21 days post-iron infusion (Ratio 1.00 (95% CI 0.99-1.00), Z-ratio=-0.95, p-value=0.43).
- There was no evidence of an increase in corrected calcium levels from 6 days post to 21 days post-iron infusion (Ratio 1.00 (95% CI 1.00-1.01), Z-ratio=0.57, p-value=0.57).
